# Acknowledgement to Reviewers of *Behavioral Sciences* in 2019

**DOI:** 10.3390/bs10010039

**Published:** 2020-01-20

**Authors:** Behavioral Sciences Editorial Office

**Affiliations:** MDPI, St. Alban-Anlage 66, 4052 Basel, Switzerland

The editorial team greatly appreciates the reviewers who have dedicated their considerable time and expertise to the journal’s rigorous editorial process over the past 12 months, regardless of whether the papers are finally published or not. In 2019, a total of 176 papers were published in the journal, with a median time to first decision of 17 days and a median time from submission to publication of 42 days. The editors would like to express their sincere gratitude to the following reviewers for their generous contribution in 2019:
A. Washburn, DavidLewko, JolantaAarts, EmmekeLiang, HuajunAderibigbe, SimonLikforman-Sulem, LaurenceAebischer, VerenaLim, Weng MarcAhmed, ZehraLing, BoAhmedien, DiaaLinks, PaulAlba De La Fuente, AnahiLisinskiene, AusraAllé, Mélissa C.Lisinskienė, AušraAllen, Michael ToddLitofsky, Norman ScottAlt, MariusLiu, YanAmsterdam, Jan GC VanLiutsko, LiudmilaAmutio, AlbertoLöchner, JohannaAnastasiu, LiviaLochtman, KatjaAnderson, JohnAELodi, ErnestoAng, Ian Yi HanLoos, EugèneAnnus, PaulLovell, GeoffAragon, OrianaLowet, KoenArribas Galarraga, SilviaLui, FaustaAseervatham, JayaLunov, VitaliiAslanidis, TheodorosMachado, CarolinaAssari, ShervinMachimbarrena, Juan M.Babcock, LauraMaciejewski, GrzegorzBadau, AdelaMagrane, DianeBadau, DanaMahmuda, Naila AlBadicu, GeorgianMajor, Sofia O.Baena-Extremera, AntonioMaki, YohkoBailey, KylieMalinowska-Cieślik, MartaBajs, Irena PandžaMalisa, MarkBalcells-Balcells, AnnaMann, Frank DBallarotto, GiuliaMannan, HaiderBanerjee, KalpitaMannino, GiuseppeBarkauskienė, RasaManuti, AmeliaBarraza, JorgeMaple, MyfanwyBarrios, MaiteMarcassa, StefaniaBasic, GoranMarks, LorenBattaglini, LucaMartin, Richard H.Baudson, Tanja GabrieleMartinez, AlejandroBaul, TushiMartínez-León, NataliaBayram-Jacobs, DurdaneMartini, DanielaBeaujean, AlexanderMartins, IanBelcher, John R.Marzoli, DanieleBelford, NishMasala, CarlaBenbow, Alison E. F.Mascitti, MarcoBendick, MarcMassart, AlainBerard, Marie-FranceMatud, PilarBerg, KristenMatud, María PilarBergold, SebastianMay, ChrisBernardi, AndreaMazilescu, Crisanta-AlinaBhochhibhoya, AmirMazur, JoannaBhuyan, RupaleemMazzone, AngelaBień, AgnieszkaMcFadden, SandraBikov, AndrásMcKinney, BrandonBlinkhorn, VictoriaMeads, CatherineBockerman, PetriMei, XiaohanBogacz-Wojtanowska, EwaMeisenberg, GerhardBogdański, PawełMeneses, Rute FBoling, Warren W.Merchant, HamidBondoc, IonelMichalska-Dudek, IzabelaBoomkens, CynthiaMignon, SylviaBorzikowsky, ChristophMikroyannidis, AlexanderBotturi, AndreaMiles, DebraBracci, EnricoMishra, JyotiBreeze, RuthMishra, AvinashBrooke, JoanneMoaddab, MahsaBrunec, IvaModenese, AlbertoBuckley, JeffreyMoè, AngelicaBuksnyte–Marmiene, LoretaMolero Jurado, María Del MarBurghardt, GordonMonteiro, DiogoBurgos-Garcia, AntonioMoon, Il SooBurlacu, SorinMoret-Tatay, CarmenCalabria, MarcoMorinaj, JuliaCalabuig-Moreno, FerranMorrissey, HanaCallegari, CamillaMortimer, MichaelCampus, GuglielmoMoustaïd-Moussa, NaïmaCaputo, FabioMucci, NicolaCaraballo, M. AngelesMuñoz Pérez, CarlosCarner, Serafin CambrayNapal, MaríaCarson, FraserNavarro, RaulCarvalho, JanaynaNavidi, UteCasais, BeatrizNemzer, Louis RCastaldi, ElisaNewbury, Joanne B.Cava, María-JesúsNewhouse, IanCava-Tadik, YaseminNewson, LisaCave, KyleNg, Qin XiangCelaya, M. LuzNg, KwokCerejeira, JoaquimNguyen, BinhCerna, MiloslavaNie, PengCerniglia, LucaNikishina, Vera BorisovnaChacón Cuberos, RamónNikolaou, Charoula KonstantiaChang, Yun-HsuanNolan, ClodaghChang, Yao-LangNosetti, LuanaChang, Yun-HsuanNounagnon Frutueux, AgbanglaChatterjee, SatabdiO’Dwyer, SiobhanChen, Liang-YuOe, MisariChenchev, IvanOlagunju, Kehinde.OluseyiChiappini, StefaniaOlteanu, AlinChiew Hong, NgOmar, Syed HarisChild, StephanieOrmsbee, FloydChoi, JihyeOsin, Evgeny N.Chokoshvili, DavitOsorio, CarlosChoudhuri, AvikOtwinowska-Kasztelanic, AgnieszkaChristopoulos, AthanasiosOuhnana, MarouaneCicero, ArrigoOwens, RheaCiorciari, JosephPaap, Kenneth R.Clarke, AlasdairPace, NelsonClaros, EdithPancotto, FrancescaClucas, RobPanek, JiriColomé, ÀngelsPapadakis, StamatisComan, EmilPapandreou, MariaComan, AlinPapousek, IlonaConnor, Helene DianaParadowski, Michał B.Constantin, CristinelPark, Jin-SungConti, DanielaParra Camacho, DavidCorreia Caeiro, CatiaParra-Meroño, María ConcepciónCothran, Dee LisaParv, LuminitaCoussens, ScottPatalas-Maliszewska, JustynaCrayen, ClaudiaPathak, DhrubaCribbet, Matthew R.Pawar, AmbarishCriss, ShaniecePearce, John A.Cvorovic, JelenaPekmezovic, TatjanaDa Luz, Felipe Q.Pereira, DoraDa Veiga, Claudimar PereiraPerenyi, AronDal Ben, MatteoPérez-Fuentes, María Del CarmenDaumiller, MartinPérez-Rueda, AlfredoDavison, KarenPhillips, Andrew J. K.De Barros Camargo, ClaudiaPietsch, StefanieDe Bruin, AngelaPilling, MichaelDe Gennaro, LuigiPinho, Maria Gabriela M.de la Haye, KaylaPinkas, AdiDe La Torre, Gabriel G.Pisoni, GalenaDe Souza, Claudia DanielePolanska, KingaDe Stefano, RosaPollak, YehudaDel Barrio Gándara, M VictoriaPonzini, GiuseppeDelgado, PauloPopa-Wagner, AurelDeligeoroglou, EfthimiosPoth, ChristianDempsey, Heidi L.Poulová, PetraDesai, AbhishekPourié, GrégoryDi Cristofori, AndreaPoza-Vilches, FátimaDi Dio, CINZIAProgler, JosephDibaba, DanielProtzko, JohnDissanayake, EllenQuam-Wickham, NancyDonizzetti, Anna RosaQuan, Stuart F.Dos Santos, Luis MiguelQuijano-López, RocíoDruica, ElenaRabinowitz, SamuelDubiel, BozenaRahman, OsmudDzambas, TamaraRajavenkatanarayanan, AkileshEconomou, AthinaRamachandran, RoshiniEdgeston, AnnaRamirez-Baena, LuciaEdler, DennisRamos Salazar, LeslieEdmondson, AmandaRamos-Vidal, IgnacioEiroa-Orosa, Francisco JoséRapp, PaulElison, JeffRaptis, George EEpstein, NurithRecchia, Daniela R.Etter, DarrylReeves, AdamEvans, Jonathan St B. T.Ren, QingzhongEverri, MarinaReybrouck, MarkFargnoli, MarioReynolds, AmyFarrand, KathleenRhein, MathiasFasko, DanielRichardson, George B.Fawcett, BarbaraRicou, MiguelFeinn, Richard S.Rivas, Carol A.Fernandez Salinero, SamuelRiza, ElenaFernández-Prados, Juan SebastiánRobinson, Mark A.Filipiak, SaraRoca, FrancescFincham, AndrewRoccella, MicheleFischer, MatthiasRodrigues, FilipeFisher, Jane E.Rodríguez, Carlos FreireFitness, JulieRodríguez, FranciscoFitzpatrick, EstherRogozea, LilianaFoulsham, TomRomero Martínez, ÁngelFranca, LuciaRozgonjuk, DmitriFreedman-Doan, CarolRuggieri, Ruggero AndrisanoFreeman, MaxRuiz-Palmero, JulioGaleazzi, Gian MariaRutkauskaite, RenataGallar, DavidSacrey, Lori-Ann R.Gander, FabianSafron, AdamGanesan, AshaSah, NirnathGarcia, AmberSáiz Manzanares, María ConsueloGarcía, OscarSalisbury, JosephGarcía-Martínez, InmaculadaSamadi, Sayyed AliGarg, KawishSamsam, MohtashemGarrett, Mario D.Sankarasubramanian, VishwanathGarrido, Juan E.Savolainen, IinaGarr-Schultz, AlexandraScandurra, CristianoGarssen, BertSchubert, Anna-LenaGarzon, XimenaSchumm, Walter R.Gasca-Salas, CarmenSegal, UmaGash, HughSegura, AdriánGavurova, BeataSegura Robles, AdrianGibbons, JeffSeifried-Dübon, TanjaGil Madrona, PedroSekulic, DamirGłąbska, DominikaSellner, JohannGómez-Urquiza, Jose L.Sembajwe, GraceGonda, XeniaSemu, Linda L.Goniewicz, KrzysztofŞerban-Oprescu, George LaurenţiuGonzález-Cabrera, JoaquínServidio, RoccoGoswami, SaheliServidio, Rocco CarmineGraham, John H.Shafer, Daniel M.Gray, SelenaShamionov, Rail M.Greco, GianpieroSharma, AmitGrimholt, Tine K.Shelley-Tremblay, JohnGriñán-Ferré, ChristianShintel, HadasGrubić Kezele, TanjaShuffrey, Lauren C.Gu, FengShust, EfratGüeita Rodríguez, JavierSienkiewicz-Małyjurek, KatarzynaGuido, GiuseppeSignorelli, MarcelloGuinjoan, MarcSimenko, JozefGusev, AlexeySimeon, James C.Gutierrez, AntonioSiniscalco, DarioGyurák Babeľová, ZdenkaŚlaski, SławomirHalstead, BrianSłomka, ArturHanć, TomaszSokal-Gutierrez, KarenHandcock, Phil J.Solovieva, YuliaHanke, StenSorensen, CarlHannon, BrendaSorfa, DavidHannonen, RiittaSoundy, AndrewHarbottle, CeliaSousa, Maria JoséHarding, RosieSpitzenstetter, FlorenceHarris, RichardȘtefan, Simona CătălinaHarrison, TeresaStetina, Birgit U.Hartanto, AndreeŠtrac, Dubravka ŠvobHasanagas, Nikolaos D.Strielkowski, WadimHawkins, Raymond CStruiksma, MarijnHeinz, MelindaSturiale, Carmelo LucioHemingway, AnnSudraba, VelgaHerzberg, Philipp Y.Sulavko, AlexeyHimmerich, HubertusSummavielle, TeresaHinz, LisaSun, Kai SingHo, Roger C.Suñer Soler, Maria RosaHo, CyrusSurguchov, AndreiHoare, DerekSutton, JillHodgson, TimSvartengren, MagnusHolbrook, JackSzathmáry, EörsHöltge, JanSzatkiewicz, Jin P.Hörnsten, ÅsaSzékely, AndreaHou, Wen-HsuanTambyraja, Sherine R.Howell, AaronTan, Shu LingHu, XiaosuTang, ShifangHuang, Yi-TingTanti, Goutam KumarHung, Li-SanTararova, OlgaHunt, JodiTassé, MarcHuta, VeronikaTaveira, Maria Do CéuIacono, DiegoTaylor, RachelInchausti, FelixTe Nijenhuis, JanInglés, CándidoTemesi, AgostonInvitto, SaraTerry, DanielIorga, MagdalenaTescasiu, BiancaIvanova, AlyonaThai, YvonneIzaguirre, AinhoaTheilmann, FlorianIzawa, Kazuhiro P.Thoma, Colleen AJack, DanaThomas, ChristianJain, SameerTitone, DebraJanikowska, GrażynaTok, SerdarJankovic, MomciloTomaževič, NinaJansen, PetraTomba, ElenaJaquess, KyleTopa, GabrielaJavanbakht, ArashTopolinski, SaschaJesulola, EmmanuelTorgersen, Eivind NessaJin, JungheeToronov, VladislavJohnson, ScottTremolada, MartaJones, StephenTrimmel, MichaelJones, TiffanyTrip, SimonaJordan, CatherineTroisi, JacopoJosé Santamaría, JuanTschacher, WolfgangKahsay, ZnabuTse, Chi-ShingKajamies, AnuTsytsarev, VassiliyKanamori, MasaoTünnermann, JanKang, Seung-WanUher, JanaKang, KyonghwaUnger, AlexanderKärkkäinen, SirpaUrbanek, ArkadiuszKastrup, BernardoUrbański, MariuszKatsounari, IoannaUrra, XabierKaye, Linda K.Urschler, David F.Kelly, DanielUsami, SatoshiKelly, NiamhUseche, Sergio A.Kelman, Ari Y.Valentino, MarioKiley-Worthington, MartheVan den Noort, MauritsKim, SeoyongVasandani, PareshKim, Byung-JikVeiga, Feliciano HenriquesKim, Jin-BomVerma, NirmalKindermann, HaraldVihman, Virve-AnneliKiyono, KenVincent, GraceKlein, JennieVisković, IvanaKochetova, Tatyana ViktorovnaVišnjić, AleksandarKoga, Patrick MariusVisser, Adriaan PhKonstantinov, VsevolodVogelaar, BartKorcz, AgataVon Wyl, AgnesKothgassner, Oswald D.Voroshilova, AnzhelikaKovac, StjepanaVrana, ScottKoven, Steven G.Wainwright, JohnKožuh, InesWalentynowicz, MartaKragness, HaleyWalker, MarvinKrajcsák, ZoltánWang, Jeff CW.Krajenbrink, TrudyWang, ShirleyKratochvíl, LukášWard, NathanKretzschmar, AndréWarms, Richard L.Kreuzpointner, LudwigWarrington, Junie PKrysinska, KarolinaWatson, Silvana Maria RussoKubota, MakiWelsh, JaneKukem, Tesfaye SemelaWenos, JeanneKulke, LouisaWest, JuliaKumar, SampathWhisenant, MeaganKyriacou, CharalambosWielki, JanuszLachmann, BerndWijnen, HermanLago, Tiffany R.Williams, IsobelLAI, Lufanna Ching-hanWilson, Joseph ALai, IvanWinings, KathyLajante, MathieuWlaź, PiotrLalueza, José LuisWojciechowski, AdamLane, Scott D.Wolniak, RadoslawLane, AndrewWong, Chi-kin KenchiLange, EwaWright, Elizabeth QuinnLara Sánchez, Amador JesúsYang, XiaoLardone, AnnaYang, BoLautrey, JacquesYang, XiaozhaoLavender, AndrewYumi, HashizumeLawson, Nicholas D.Zadra, CinziaLee, Paul H.Zakrocka, IzabelaLehner, MałgorzataZhang, MelvynLeicht, Kevin T.Zhao, TingLeightley, DanielZhu, LinLeón del Barco, BenitoZielinski, SlawomirLewin, EyalZubiaur, MartaLewis, Ghislaine L.


